# Evaluating the Potential of *Plukenetia volubilis* Linneo (Sacha Inchi) in Alleviating Cardiovascular Disease Risk Factors: A Mini Review

**DOI:** 10.3390/ph16111588

**Published:** 2023-11-09

**Authors:** Izzat Zulhilmi Abd Rahman, Nur Syahidah Nor Hisam, Amilia Aminuddin, Adila A. Hamid, Jaya Kumar, Azizah Ugusman

**Affiliations:** 1 Department of Physiology, Faculty of Medicine, Universiti Kebangsaan Malaysia, Jalan Yaacob Latif, Kuala Lumpur 56000, Malaysia; izzat_zulhilmi@ukm.edu.my (I.Z.A.R.); p104164@siswa.ukm.edu.my (N.S.N.H.); adilahamid@ppukm.ukm.edu.my (A.A.H.); jayakumar@ukm.edu.my (J.K.); 2 Programme of Biomedical Science, Centre for Toxicology & Health Risk Studies, Faculty of Health Sciences, Universiti Kebangsaan Malaysia, Jalan Raja Muda Abdul Aziz, Kuala Lumpur 50300, Malaysia

**Keywords:** antioxidant, cholesterol, diabetes, inflammation, obesity, *Plukenetia volubilis* Linneo, sacha inchi

## Abstract

*Plukenetia volubilis* Linneo or Sacha Inchi (SI), a traditional natural remedy indigenous to Peru and Brazil, has garnered global attention due to its exceptional nutritional composition. Its protective effects against various non-communicable diseases, notably cardiovascular disease (CVD), have become a subject of interest in recent research. This comprehensive review summarizes the existing evidence from 15 relevant articles concerning the impact of SI on common CVD risk factors, including dyslipidemia, obesity, diabetes, and hypertension. The relevant articles were derived from comprehensive searches on PubMed, Scopus, Google Scholar, and Web of Science using predefined criteria and keywords related to the topic. Overall, SI demonstrated positive effects in attenuating dyslipidemia, obesity, diabetes, and hypertension. The multifaceted mechanisms responsible for the protective effects of SI against these CVD risk factors are primarily attributed to its antioxidative and anti-inflammatory properties. While preclinical studies dominate the current scientific literature on SI, there are limited clinical trials to corroborate these findings. Therefore, future well-designed, large-scale randomized clinical trials are highly recommended to establish the efficacy of SI and determine its optimal dosage, potential drug and food interactions, and practical integration into preventive strategies and dietary interventions for the high-risk populations.

## 1. Introduction

### 1.1. Plukenetia volubilis Linneo

*Plukenetia volubilis* Linneo, also known as sacha inchi (SI), Inca inchi, mountain peanut, and Inca nut is a perennial plant belonging to the Europhorbiaceae family. Native to the Peruvian and Northwestern Brazil jungle [1], the Europhorbiaceae family comprises 300 genera and 7500 species [2]. SI naturally thrives in the rainforests of the Americas, specifically at altitudes ranging from 200 to 1500 m [3]. However, it is now commercially cultivated in Asian countries such as Thailand, China, and Vietnam, as well as in Central and South America, because of its exceptional nutritional values [4]. SI has a long-standing traditional use for treating joint problems and relieving muscle pain [5], and providing skincare benefits such as moisturization, wound healing, insect bite treatment, and combating skin infections [6]. The various components of the entire SI plant, including seeds, kernels, and leaves, have significant economic potential for commercial exploitation [7].

The different parts of the SI plant contain various active compounds that contribute to its medicinal and nutritional properties. The seeds of SI are the most widely used part of the plant. They are a rich source of essential fatty acids, particularly omega-3 (ω-3) fatty acids such as α-linolenic acid (ALA). The seeds also contain omega-6 (ω-6) and omega-9 (ω-9) fatty acids [8]. In addition, they are abundant in tocopherols (α- and γ-tocopherols), flavonoids, phytosterols (stigmasterol, β-sitosterol, and campesterol), lignans, and phenyl alcohols [9]. SI oil is extracted from the seeds and is known for its exceptional nutritional profile. It is primarily composed of unsaturated fatty acids, including ω-3, ω-6, and ω-9 fatty acids. The oil is particularly high in ALA, which contributes to its ω-3 content. It also contains tocopherols, an antioxidant that helps protect the oil from oxidation [10]. SI oil has a favorable ratio of ω-3 to ω-6 fatty acids, making it a valuable addition to a healthy diet [11].

The leaves of the SI plant contain a variety of bioactive compounds, including phenolic compounds, flavonoids, and terpenoids. These compounds contribute to the antioxidative and anti-inflammatory properties of the leaves [7,12]. The leaves also contain chlorophyl, which provides a vibrant green color and has detoxifying properties [13]. Although less extensively studied, the roots of SI are shown to contain alkaloids, tannins, flavonoids, leptins, saponins, and other bioactive compounds [14]. The specific composition of the roots may vary and require further research to fully understand their nutritional and medicinal properties. Similarly, there is limited information on the SI shell composition. A small amount of lipid and a considerable amount of phenolic content in the shell has been reported [7]. The composition of SI plant parts can be influenced by factors such as growing conditions, environmental factors, and processing methods. These variations can affect the nutrient content and overall composition [15,16]. However, SI, in its various forms, offers a rich array of nutrients and bioactive compounds that contribute to its potential health benefits, including the potential to reduce risk factors associated with cardiovascular diseases (CVD).

### 1.2. Cardiovascular Diseases

CVD are chronic conditions affecting the heart and blood vessels, such as coronary artery disease (CAD), hypertension, stroke, and peripheral artery disease. They progress slowly and often remain asymptomatic for an extended period of time [17,18]. CVD remains the leading cause of death worldwide, accounting for 32% of global mortality in 2019, resulting in an estimated 17.9 million deaths [19]. This number is projected to rise to approximately 23 million by 2030 [20]. Furthermore, CVD has significant economic and social consequences. CVD have a negative effect on the patients’ well-being, influencing their job performance and effectiveness, and resulting in significant financial setbacks, which can be interpreted as a reduction in human capital [21,22]. 

The economic loss due to workplace absenteeism due to chronic disease-related functional limitations among the working-age population is estimated to be approximately 4.95 billion per year [23]. According to the American Heart Association, the total expenses related to CVD in the United States in 2019 were estimated to be approximately USD 351.2 billion. This includes costs related to hospitalization, medications, procedures, and other medical expenses associated with the diagnosis and treatment of CVD [24]. While recent studies indicate a significant decrease in the prevalence of CVD in developed countries due to major advancements in CVD prevention and treatment [25], there has been an increasing trend of CVD in developing nations [26]. This rise can be attributed to the higher prevalence of CVD risk factors such as diabetes, hypertension, hypercholesterolemia, overweight/obesity, and smoking, which contribute to the development of atherosclerosis, CAD, and stroke [27,28]. 

### 1.3. The Role of Oxidative Stress and Inflammation in CVD

There is a significant body of evidence linking CVD and its risk factors to oxidative stress and inflammation [29]. Oxidative stress refers to an imbalance between the production of reactive oxygen species (ROS) and the body’s ability to neutralize them, leading to cellular damage. Inflammation, on the other hand, is the body’s immune response to harmful stimuli, such as infection or injury. A wide array of evidence strongly supports the pivotal role of oxidative stress and inflammation in the pathogenesis of CVD by contributing to vascular endothelial dysfunction [30].

Oxidative stress and inflammation are the primary catalysts for vascular endothelial dysfunction. The development of endothelial dysfunction is characterized by a reduction in the synthesis and/or bioavailability of nitric oxide (NO) [31]. This impairment can be attributed to several oxidative enzyme systems, including NADPH oxidase, xanthine oxidase, uncoupled endothelial nitric oxide synthase (eNOS), cyclooxygenases, lipoxygenases, and myeloperoxidases, which produce superoxide anion and contribute to vascular oxidative stress. This, in turn, leads to the deactivation of NO and subsequent endothelial dysfunction [32,33].

Numerous traditional risk factors for CVD, such as smoking, hypercholesterolemia, hypertension, obesity, and diabetes, are closely associated with endothelial dysfunction. These risk factors foster chronic inflammation, resulting in an increase in vasoconstrictive and prothrombotic substances, a decrease in antithrombotic factors, and abnormal vasoreactivity, collectively increasing the risk of cardiovascular events [34]. Elevated levels of pro-inflammatory cytokines like tumor necrosis factor-alpha (TNF-α), interleukin-1beta (IL-1β), interleukin-6 (IL-6), and interferon gamma (IFN-γ) have been detected in vascular endothelial dysfunction, primarily due to the activation of the nuclear factor-kappa B (NF-κB) pathway [35]. NF-κB serves as a crucial transcription factor that promotes CVD by stimulating the transcription of pro-inflammatory, pro-adhesion, and pro-oxidant genes. The NF-κB pathway is activated by various stimuli, including inflammatory cytokines, ROS, lipids, and mechanical forces acting on the vascular endothelium [36].

Moreover, endothelial dysfunction serves as a precursor to atherosclerosis, which is a primary underlying cause of CVD. The mechanisms contributing to atherosclerosis include the activation of pro-inflammatory signaling pathways, cytokine expression, and increased oxidative stress [37,38]. Risk factors for CVD, such as dyslipidemia, hypertension, and diabetes, synergistically induce endothelial dysfunction, leading to endothelial inflammation, macrophage differentiation, foam cell formation, platelet adhesion, and thrombus formation in the atheroma. These processes result in arterial narrowing and an increased risk of heart attack and stroke [39].

Thus, oxidative stress, inflammation, and vascular endothelial dysfunction are interconnected and have significant implications in the development and progression of CVD. In the context of CVD, persistent inflammation creates a vicious cycle that drives the progression of CVD-related complications [40]. Hence, targeting oxidative stress and inflammatory pathways may provide opportunities for interventions aimed at reducing CVD burden and improving cardiovascular health. 

### 1.4. The Potential of SI for Cardiovascular Health

Recently, the utilization of natural products for the management and prevention of CVD has gained significant attention. Natural products derived from plants and other natural sources possess various bioactive compounds with antioxidative and anti-inflammatory effects that have the potential to promote cardiovascular health [41]. Several recent reviews have explored the nutritional compositions, physicochemical characteristics, extraction techniques, biological activities, and potential uses of SI for human health [7,14,15,42]. Although there have been initial studies and reviews concentrating on the health benefits of SI, there is currently a lack of a comprehensive review that emphasizes the potential of SI in reducing risk factors for CVD. Furthermore, as new research continues to emerge, there is a pressing need for an updated synthesis of findings related to SI and its benefits in the context of CVD.

Given the global burden of CVD and the increasing interest in plant-based preventive and therapeutic solutions, comprehending the potential of SI could open the door to innovative dietary recommendations and potential therapeutic treatments. In the following sections, we explored the potentials of SI in addressing prevalent modifiable risk factors of CVD, with a particular focus on its effects on dyslipidemia, obesity, diabetes, and hypertension. Considering the multifaceted and interconnected nature of these four common risk factors, addressing them has the potential to lead to substantial reductions in overall CVD risk [43]. Each section provides a comprehensive analysis, supported by the latest scientific evidence, and offers an in-depth perspective on the contribution of SI in mitigating risk factors associated with CVD.

## 2. Literature Search

The relevant literature on this topic was searched across four primary electronic databases, namely PubMed, Scopus, Google Scholar, and Web of Science, from 2010 to 2023 using the search string ‘*Plukenetia volubilis* Linneo, Sacha Inchi’ AND (antioxidant OR inflammation OR cholesterol OR obesity OR diabetes OR hypertension). Only original research articles aimed at elucidating the effects of SI on CVD risk factors, including hypertension, dyslipidemia, obesity, and diabetes in preclinical and clinical settings were included. The literature search yielded 15 relevant articles. In presenting the extracted data, the information was systematically organized into sections detailing the experimental model used, the preparation and dosage of SI, the outcomes, and the potential mechanisms of action. This structured approach ensured comprehensive coverage and clear characterization of the topic area.

## 3. Effects of SI on CVD Risk Factors

### 3.1. Effects of SI on Dyslipidemia

Within the spectrum of CVD risk factors, dyslipidemia has been identified as the most potent contributor [44]. Elevated serum levels of total cholesterol (TC), LDLc, and triglycerides (TG), or a decrease in serum high-density lipoprotein cholesterol (HDLc) levels are well-established risk factors for CVD [45]. The beneficial effects of SI on cholesterol levels have been confirmed in animal and clinical studies. Obese rats fed 2.5 mL of SI emulsion oil with different ω-3 content (0.2 g and 0.5 g ω-3/day) for eight weeks showed decreases in TC, TG, and LDLc levels, and increases in HDLc levels [46]. The treatment of patients with hypercholesterolemia with 5 or 10 mL of SI oil (contains 2 g and 4 g ω-3/day, respectively) for 16 weeks also caused a significant reduction in their total cholesterol and LDLc and increment in their HDLc levels [47]. 

SI oil is also helpful in preventing dyslipidemia in healthy individuals. Consumption of 10 or 15 mL of SI oil by healthy individuals for 16 weeks was found to significantly reduce their serum TC and LDLc levels and increase their HDLc levels [48]. In a randomized crossover clinical trial, the consumption of 15 mL SI oil alongside a high-fat meal reduced the postprandial increase in TC levels and the inflammatory marker interleukin- 6 (IL-6) in metabolically healthy men. However, no significant differences were observed in their HDLc and TG levels, which is most likely due to the short duration of the intervention [49]. In contrast, SI oil intake with a high-fat meal reduced the postprandial increase in IL-6 but failed to reverse the postprandial cholesterol increase in metabolically unhealthy men. This shows that the effect of SI on postprandial lipid levels following a high-fat meal depends on the individual’s metabolic status [49]. 

SI exerts antihyperlipidemic activity in vitro, mainly via enzymatic inhibitory reaction [50]. The 3-hydroxy-3-methylglutaryl-coenzyme A (HMG-CoA) reductase is a rate-controlling enzyme in the mevalonate pathway of cholesterol biosynthesis. HMG-CoA reductase inhibitors, such as statins, have long been used to efficiently treat hypercholesterolemia. However, its multisystemic adverse effects necessitate the discovery of plant-based HMG-CoA reductase inhibitors with minimal side effects [51,52]. An in vitro study showed that 125 µg/mL of SI nutshell hot water extract inhibited HMG-CoA reductase activity by 65% [53]. Contrary to the mechanism of action of statins, SI nutshell extract exhibits a precise and effective non-competitive inhibition pattern [53,54]. In addition, there was a 38.1% reduction in cholesterol esterase activity using a similar SI extract concentration [53]. The inhibition of cholesterol esterase activity interferes with cholesterol absorption and transport into enterocytes, which contributes to the lipid-lowering effect of SI [55]. However, SI baby nut and leaf hot water extracts and SI nut oil did not show any HMG-CoA reductase or cholesterol esterase inhibitory effects [53].

**Figure 1:**
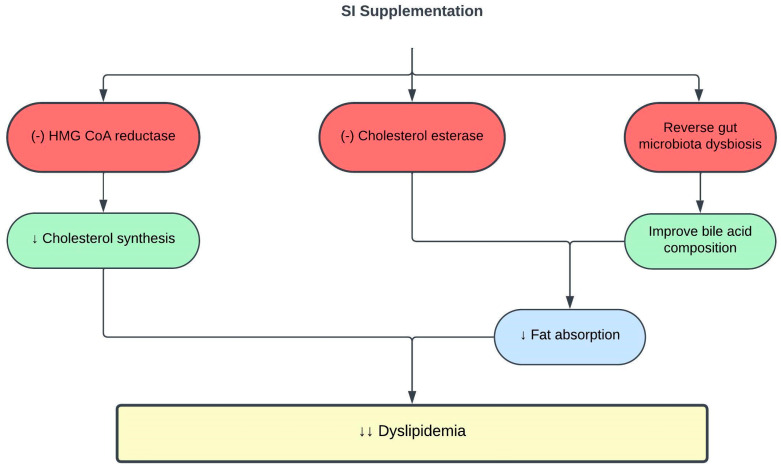
Protective effects of SI on dyslipidemia. (-), inhibit; ↓, decrease; HMG-CoA, 3-hydroxy-3-methylglutaryl-coenzyme A.

Recent studies have identified a close relationship between the gut microbiome and dyslipidemia [56,57]. High dietary fat induces dysbiosis of the gut microbiota, resulting in lipid dysmetabolism [58]. SI oil consumption reversed gut microbiota dysbiosis in high-fat diet (HFD)-fed rats, thus improving their TC, TG, and LDLc levels [59]. One of the mechanisms is through the influence of the gut microbiota metabolome on bile acid composition. Bile acids regulate hepatic lipid metabolism by facilitating lipid absorption [60,61]. HFD altered gut microbiota metabolome and bile acid composition in the small intestine, leading to elevated levels of taurocholic acid (TCA), taurochenodeoxycholic acid (TCDCA), cholic acid, and glycocholic acid, which contribute to lipid dysmetabolism and hyperlipidemia. Supplementation with 0.5–1.5 mL/kg SI oil for eight weeks reversed the changes in gut microbiota metabolome and bile acid composition, and improved lipid dysmetabolism in HFD-fed rats [59]. Table 1 and Figure 1 summarize the current knowledge on the protective effects of SI on lipid metabolism and dyslipidemia. 

### 3.2. Effects of SI on Obesity

CVD is associated with obesity and high visceral fat deposition [62]. SI exerts anti-obesity effects in vivo. High-fat diet (HFD)-fed Sprague Dawley rats supplemented with 0.5–1.5 mL/kg SI oil for eight weeks showed significant reduction in their adipocyte size, hepatic steatosis, and inflammation. This is associated with increased hepatic lipase expression following SI oil supplementation [59]. Hepatic lipase is an essential lipolytic enzyme that facilitates lipoprotein uptake in the de novo lipid synthesis pathway [63]. Furthermore, SI oil suppressed the production of *lysophosphatidylcholine* (LysoPC) and lysophosphatidylethanolamine (LysoPE) [59], which have been identified as pro-inflammatory phospholipids found in patients with hyperlipidemia and HFD-induced obesity [64]. In addition, SI oil decreased the expression of hepatic phosphatidylglycerol phosphate synthase 1 (PGS1) in HFD-fed rats [59]. PGS1 catalyzes the production of phosphatidylglycerol, which is a potent inhibitor of lipolysis [65]. Therefore, the inhibition of PGS1 by SI oil reduced phosphatidylglycerol production and improved lipolysis [59]. 

Pancreatic lipase has been identified as a key enzyme in systemic lipid digestion and absorption [66]. Its inhibition has gained significant interest as an efficient method to reduce obesity [67]. Interestingly, SI meal-derived peptides, a by-product generated from SI oil, exhibited strong pancreatic lipase inhibitory activity, hypothetically by competitive binding to the pancreatic lipase catalytic sites [68]. Pancreatic lipase inhibitory peptides were previously demonstrated to reduce the amount of intracellular fat accumulations by neutralizing ROS production in oleic acid-induced HepG2 cells [68]. Meanwhile, the SI protein isolate showed high antioxidative activity, which might contribute to its pancreatic lipase inhibitory effect [69]. A similar lipase inhibition pattern was also observed with SI husk aqueous ethanol extract [70]. The lipase inhibitory effect of SI may be attributed to the synergistic actions of its various phenolic and active peptide compounds [70].

Obesity leads to complications such as dyslipidemia and CVD through mechanisms involving oxidative stress, low-grade inflammation, and cellular hypoxia [71]. Oxidative stress promotes lipid and protein oxidation [72], as evidenced by the production of the end-products; malondialdehyde (MDA) and advanced protein oxidation products (AOPP) [73,74,75]. Treatment of obese rats with the emulsion of SI oil (2.5 mL, contains 0.25 g and 0.5 g ω-3/day) reduced MDA and AOPP levels in the rat serum [46]. SI oil emulsion also enhanced antioxidant capacity by stimulating the activity of the antioxidant enzyme catalase [46,76]. Furthermore, SI oil emulsion attenuated inflammation in obese rats, as evidenced by reduced levels of the proinflammatory cytokines IL-6 and tumor necrosis factor-α (TNF-α) [46]. Collectively, the findings showed that the antioxidative and anti-inflammatory activities of SI contribute to its anti-obesity effect. 

**Figure 2:**
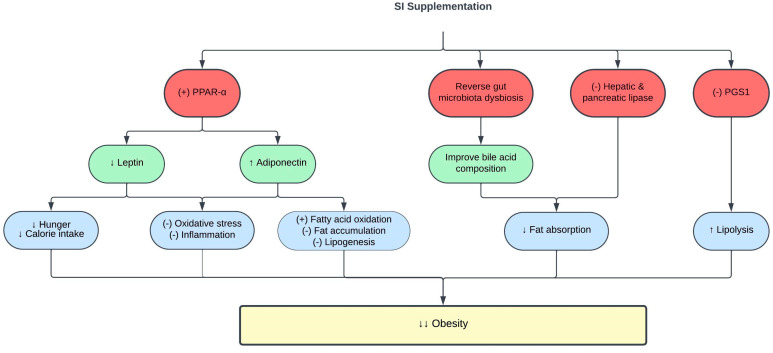
Protective effects of SI on obesity. (-), inhibit; (+), stimulate; ↑, increase; ↓, decrease; PGS1, phosphatidylglycerol phosphate synthase 1; PPAR-α, peroxisome proliferator-activated receptor alpha.

The pro-inflammatory state in obesity is associated with changes in adipokine release by adipose tissues, such as leptin and adiponectin [77,78]. Leptin regulates body fat composition by augmenting energy expenditure and inhibiting appetite [79]. However, leptin resistance and hyperleptinemia in obesity promote hunger, increase food consumption, and induce inflammation [80]. Meanwhile, adiponectin is an anti-inflammatory adipokine, and its level is reduced in obesity [81]. Interestingly, the emulsion of SI oil reduced leptin levels and increased adiponectin levels in obese rats [46]. Increased adiponectin levels are also responsible for the hypolipidemic effect of SI, as adiponectin activates lipoprotein lipase to degrade circulating TG [82]. The positive effects of SI oil on leptin and adiponectin levels were mediated via increased expression of peroxisome proliferator-activated receptor alpha (PPAR-α), which is a crucial transcription factor that regulates fatty acid metabolism and oxidative stress [76]. Table 2 and Figure 2 summarize the current knowledge on the protective effects of SI on obesity. Exploring the impact of SI supplementation on body mass index, body fat composition, and basal metabolic rate in individuals with obesity presents an intriguing opportunity since there has been no prior research delving into this subject.

### 3.3. Effects of SI on Glucose Metabolism and Diabetes

Hyperglycemia is the hallmark of diabetes mellitus, which is also a risk factor for CVD [83]. The inhibition of α-glucosidase, α-amylase, and dipeptidyl peptidase (DPP-IV) is a common method used to assess the potential antidiabetic properties of natural products. α-glucosidase inhibitors block enzymes such as glucoamylase, sucrase, maltase, and isomaltase found at the brush border of the intestinal epithelium. This inhibition prevents the absorption of carbohydrates in the small intestine, reducing postprandial hyperglycemia [84]. On the other hand, α-amylase inhibitors hinder the breakdown of α-(1–4)-d-glucosidic linkages in starch, thereby decreasing carbohydrate digestion and absorption in the gastrointestinal tract and consequently lowering blood glucose levels [85]. DPP-IV inhibitors block the degradation of incretin hormones, specifically glucagon-like peptide-1 (GLP-1) and glucose-dependent insulinotropic polypeptide (GIP). Increased GLP-1 and GIP levels stimulate insulin release, inhibit glucagon secretion, and delay gastric emptying, thus improving postprandial glucose levels.

It has been reported that 0.035 mg/mL of SI husk and shell extracts exhibited strong α-glucosidase and α-amylase inhibitory activities [70]. Correspondingly, 25 µg/mL of SI essential oil exhibited robust α-amylase inhibition [86]. The potent α-glucosidase and α-amylase inhibitory effects are linked to the phenolic content in SI [87,88,89,90]. Furthermore, SI meal-derived peptides demonstrated potent DPP-IV inhibitory activity in vitro, which was further validated with increased glucose consumption by palmitic acid-induced insulin resistant HepG2 cells [91]. It is important to emphasize that α-amylase, α-glucosidase, and DPP-IV inhibitory assays serve as valuable screening tools to evaluate the antidiabetic potential of natural products. However, it is crucial to complement these results with further in vivo studies to validate their antidiabetic activity.

The most employed in vivo model for inducing diabetes in laboratory animals involves the chemical ablation of pancreatic β-cells using streptozotocin (STZ). STZ functions as a toxic glucose analog that selectively accumulates within pancreatic β-cells via the GLUT2 glucose transporters located on the plasma membrane. Once taken up by β-cells, STZ initiates oxidative stress and DNA alkylation, ultimately leading to pancreatic β-cell necrosis, reduced insulin production, and hyperglycemia [92,93,94]. Furthermore, it is important to note that GLUT2 transporters are not limited to pancreatic β-cells but are also found in the epithelial cells of the kidneys and hepatocytes. Therefore, the administration of STZ may lead to kidney and liver toxicity, in addition to its capacity to harm pancreatic β-cells [95]. A single, high dose of STZ injection induces pancreatic β-cell damage and diabetes in rats, mimicking type 1 diabetes [96]. A low dose of STZ injection combined with HFD to induce hyperglycemia and insulin resistance is a method to mimic type 2 diabetes in rats [97].

The treatment of type 2 diabetic rats with 0.5–2 mL/kg of SI oil for five weeks significantly reduced fasting blood glucose levels and improved insulin sensitivity indices and glucose tolerance in a dose-dependent manner [98]. Insulin plays a pivotal role in regulating glucose metabolism by activating the insulin receptor substrate (IRS)/phosphatidylinositol 3-kinase (PI3K)/protein kinase B (Akt) signaling pathway. The dysfunction of insulin receptors (IR) and subsequent impairment of downstream signaling are critical factors in the onset of insulin resistance [99]. This condition, which is characterized by the inability of cells to respond effectively to insulin, contributes to heightened hepatic gluconeogenesis and glycogenolysis. Ultimately, these processes culminate in elevated blood sugar levels, leading to hyperglycemia in individuals with diabetes [100]. SI enhances hepatic insulin sensitivity by downregulating IR-β and stimulating IRS-1 and Akt. Moreover, SI also inhibited the activities of glucose-6-phosphatase (G-6-Pase) and phosphoenolpyruvate carboxykinase-1 (PCK-1) in the liver of diabetic rats [98]. Reduced G-6-Pase and PCK-1 levels suppress hepatic gluconeogenesis and enhance glycogenesis, thereby reducing blood glucose levels [101]. The bioactive compounds present in SI, including ω-3 fatty acids, β-sitosterols, and flavonoids, contribute to its insulin sensitizing effect [98]. 

Extensive studies have shown the role of intestinal microflora in various stages of diabetes progression [102,103]. An intimate relationship exists between gut microbiota dysbiosis and diabetes [104,105,106]. Pathogenic bacteria in patients with diabetes were elevated in parallel with pro-inflammatory cytokines such as IL-6. These findings denote the involvement of gut microbiota dysbiosis and inflammation in the pathogenesis of diabetes. Interestingly, 400 mg/kg SI leaf water extract treatment for six weeks exerted prebiotic activity in type 1 diabetic rats by diversifying beneficial intestinal bacteria, primarily Akkermansia, Parabacteroides, Bacteroides, and Alloprevotella, while simultaneously suppressing diabetes-related bacteria, including Lactobacillus, Ruminococcaceae, Ruminiclostridium, and Oscillibacter [107]. The amelioration of gut microbiota dysbiosis with SI treatment led to a reduction in blood glucose levels, and improved glucose tolerance and insulin resistance in diabetic rats [107]. 

In a clinical trial, the addition of 15 mL SI oil to a high-fat breakfast attenuated postprandial hyperglycemia and improved insulin sensitivity in healthy individuals with higher baseline triglycerides and glycemic response. In addition, these individuals also showed increased sirtuin 1 (SIRT-1) gene expression in their peripheral blood mononuclear cells 4 h postprandially [108]. The SIRT-1 gene plays a pivotal role in glucose metabolism, and its expression is downregulated in people with obesity and insulin resistance [109,110]. Hence, SI oil improves glycemic control and insulin sensitivity by enhancing SIRT-1 expression. However, to date, there is no clinical trial involving SI supplementation in patients with diabetes.

**Figure 3:**
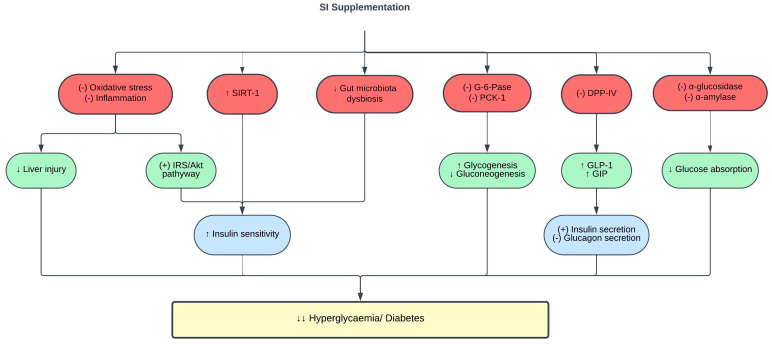
Protective effects of SI on glucose metabolism and diabetes. (-), inhibit; (+), stimulate; ↑, increase; ↓, decrease; Akt, protein kinase B; DPP-IV, dipeptidyl peptidase IV; G-6-Pase, glucose-6-phosphatase; GIP, glucose-dependent insulinotropic polypeptide; GLP-1, glucagon-like peptide-1; IRS, insulin receptor substrate; PCK-1, phosphoenolpyruvate carboxykinase-1; SIRT-1, sirtuin-1.

Diabetes leads to target organ damage, and the liver is one of the target organs of diabetes complications [111]. SI oil supplementation protected the liver from diabetes-induced liver damage, as evidenced by reduced serum alanine transaminase and aspartate transaminase (AST) levels, and improved hepatic histopathological changes [98]. The underlying mechanisms of such findings were attributed to the antioxidative and anti-inflammatory effects of SI, as treatment with SI oil reduced the oxidative stress marker (MDA), enhanced the antioxidant enzyme activity (superoxide dismutase, catalase and glutathione peroxidase) and reduced the inflammatory markers (TNF-α and IL-6) in the liver of diabetic rats [98]. However, to date, no studies have investigated how SI impacts other diabetes-related target organ damage, such as those affecting the kidneys, heart, and blood vessels. A summary of the protective effects of SI on glucose metabolism and diabetes is shown in Table 3 and Figure 3. 

### 3.4. Effects of SI on Blood Pressure

Extensive evidence has consistently established a linear relationship between elevated blood pressure and the risk of CVD [112]. To date, few studies have been conducted to study the blood pressure-lowering effect of SI. Only one clinical trial was conducted to determine the effect of SI on blood pressure in human subjects. Gonzales et al. [48] revealed that clinically healthy adults who consumed 10 and 15 mL of SI oil for four months had reduced systolic and diastolic blood pressure. The reduction in blood pressure was suggested to be due to the LDL-lowering effect of SI oil. Increased LDL levels have been linked to the pathophysiology of hypertension [113,114,115,116]. However, there is no clinical trial involving SI supplementation in patients with hypertension.

The renin angiotensin aldosterone system regulates blood pressure and body fluid homeostasis. Overactivation of this system is implicated in the pathogenesis of hypertension; hence, angiotensin-converting enzyme (ACE) inhibitors are commonly prescribed to treat hypertension [117]. In vitro evaluation using 0.013 mg/mL SI husk and shell extracts demonstrated ACE inhibitory activities [70]. Similarly, SI protein hydrolysates showed strong ACE inhibitory activity at a concentration of 98 µg/mL [118]. This ACE inhibitory effect is correlated with the high phenolic content in SI, especially in the shell [70,119]. 

Individuals with hypertension may have faulty renal handling of calcium, particularly due to upregulation of the calcium signaling pathway. This leads to increased expression of L-type calcium channels (LTCC) that allow a massive calcium ion influx into the vascular smooth muscle cells, causing vasoconstriction and increasing the blood pressure. In addition, sodium retention and plasma volume expansion have been identified as precursor of hypertension [120]. The Na^+^/K^+^-ATPase pump is essential for maintaining the electrochemical gradient of sodium across the cell membrane. In hypertension, the expression of Na^+^/K^+^-ATPase is decreased, which contributes to sodium retention and plasma volume expansion [121]. SI shell extract (SISE) exerts its antihypertensive effect in spontaneous hypertensive rats (SHR) and high-salt-diet-fed Wistar-Kyoto (WKY) rats by restoring the expression of LTCC and Na^+^/K^+^-ATPase, thus maintaining calcium and sodium homeostasis [122]. 

Oxidative stress and chronic inflammation are involved in the pathogenesis of hypertension [123]. NO produced by eNOS is an important vasoactive molecule that modulates vascular functions and blood pressure. Continuous release of superoxide exceeding the endogenous antioxidant capacity reduces NO bioavailability [124,125,126]. Reduced NO levels lead to endothelial dysfunction, impaired vasodilation, and elevated blood pressure [127]. SISE decreased the blood pressure of SHR and WKY rats fed a high-salt diet by reducing oxidative stress and inflammation, and increasing the expression of eNOS and NO. Concurrently, the level of 5-methyltetrahydrofolate (5-MTHF), an active form of di- and tetrahydrofolic acid, was found to be increased with SISE treatment [122]. 5-MTHF maintains NO bioavailability by suppressing superoxide production [128]. 

**Figure 4:**
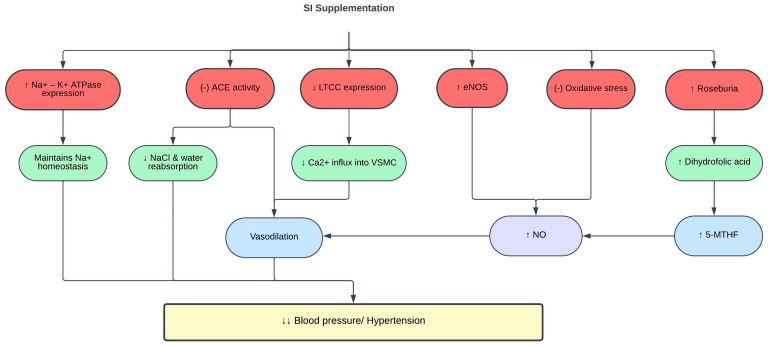
Protective effects of SI on blood pressure. (-), inhibit; ↑, increase; ↓, decrease; 5-MTHF, 5-methyltetrahydrofolate; ACE, angiotensin-converting enzyme; Ca^2+^, calcium ions; eNOS, endothelial nitric oxide synthase; K^+^, potassium ions; LTCC, L-type calcium channels; Na^+^, sodium ions; NaCl, sodium chloride; NO, nitric oxide; VSMC, vascular smooth muscle cells.

Multiple studies have linked abnormal gut microbiota to the pathogenesis of hypertension [129,130]. Gut microbiome dysbiosis or imbalance of Firmicutes/Bacteroidetes (F/B) ratio is often correlated with various pathological conditions, including hypertension [131,132]. The gut microbiota of patients and animals with hypertension also showed reduced levels of beneficial bacteria such as Roseburia, and increased levels of harmful bacteria such as Prevotella [133]. One of the mechanisms underlying the antihypertensive effect of SISE is through reshaping of the gut microbiota and metabolome, in which SISE improved the prevalence of Roseburia and dihydrofolic acid levels in the gut and normalized the F/B ratio [122]. A summary of the effects of SI on blood pressure and hypertension is shown in Table 4 and Figure 4. 

## 4. Safety of SI

Food plants that are frequently consumed by humans often contain significant secondary metabolites, which can be harmful if consumed in excess [134]. While not always harmful, specific antinutritional components can lead to adverse effects by interfering with the digestion and absorption of essential macro- and micronutrients [135]. A study reported that raw SI seed consumption induced mild to severe toxicity in humans, which was attributed to the phytotoxin content, namely alkaloids, lectins, and saponins, in raw SI seeds [14]. Srichamnong et al. discovered a very mild toxic effect of fresh SI seeds on hepatic stellate cells [136]. Phytotoxins are relatively unstable under heat [136]; therefore, roasting the raw parts of the SI plant before consumption is essential to avoid any potential health risks. 

Conversely, no morbidity or mortality was observed in acute (2000 mg/kg) and subchronic (50, 250, and 500 mg/kg for 90 days) toxicity studies and genotoxicity evaluation of rats and mice fed with SI seed powder [137]. Similarly, another oral toxicity study in rats and mice demonstrated that the consumption of 0.5 mL/kg of SI oil for 60 days was harmless, and the median lethal dose of 50 (LD50) was estimated to be more than 37 g/kg body weight [138]. Meanwhile, the consumption of 10 and 15 mL of SI oil for 16 weeks by healthy human subjects did not alter their hepatic and kidney functions, inflammatory biomarkers, and hemoglobin levels [48]. 

Despite its good safety profile, there was a rare, isolated case of occupational allergic rhinoconjunctivitis and bronchial asthma related to exposure to SI seeds [139]. Even though there were no more related cases documented, further investigation on SI allergenicity is warranted. Nausea is the most common side effect associated with SI oil consumption, which is most likely due to its unpleasant taste [48]. It is advisable to consume SI oil with food or salad for better taste. Moreover, other complaints related to gastrointestinal discomfort [48] have been reported, consistent with the previously documented minor side effects of fish oil containing ω-3 fatty acids [140]. Based on the available information, the adverse events reported by the subjects were not major and were unlikely to have a significant impact on their overall health. However, further studies are necessary to understand any potential chronic toxicity effects of SI. Furthermore, conducting a study to explore the potential combination of SI with other medications to mitigate its toxic or synergistic effects is a valuable endeavor. These discoveries would also be beneficial in considering SI as a viable dietary supplement. 

## 5. Strength, Limitations, and Future Directions

This comprehensive review provides a holistic and multifaceted understanding of SI and its therapeutic potential in addressing major CVD risk factors including dyslipidemia, obesity, diabetes, and hypertension. However, a limitation of this review is the scarcity of large-scale clinical trials on SI. Most of the findings are based on preclinical research or small-scale clinical trials. While these provide valuable insights, they may not always be directly applicable to broader, more diverse populations. Therefore, large-scale randomized clinical trials are imperative to establish the efficacy and safety of SI in mitigating CVD risk factors. Moreover, the potential for chronic toxicity of SI deserves thorough exploration. As with many natural products, prolonged SI usage may have adverse effects that remain to be fully understood. Chronic toxicity studies will not only ensure the safety of SI for long-term consumption, but also provide insights into its optimal dosage and usage patterns.

Additionally, considering the complex treatment regimen that patients with CVD frequently adhere to, understanding how SI interacts with other medications is vital. Uncovering any synergistic or antagonistic effects it may have when combined with other drugs can have significant clinical implications. This knowledge would assist healthcare professionals in prescribing SI, ensuring its seamless integration with existing treatment plans while preventing unfavorable interactions. Furthermore, mechanistic studies will aid in unraveling the underlying molecular pathways responsible for the cardiovascular benefits of SI. Investigations into optimal dosages, potential interactions with drugs and food, and applications in clinical settings will pave the way for the practical integration of SI into preventive strategies and dietary interventions for the high-risk population.

## 6. Conclusions

This review emphasizes the significant potential of SI as a natural remedy for mitigating CVD risk factors. The presence of beneficial fatty acids, antioxidants, and anti-inflammatory compounds in SI underscores its capacity to positively impact lipid profiles, blood glucose levels, adipogenesis, and blood pressure regulation. These encouraging effects position SI as a viable dietary supplement and complementary therapy in the battle against CVD. However, most of the data are obtained from preclinical studies. Consequently, it is highly recommended that large-scale randomized clinical trials be conducted in the future to establish the efficacy of SI.

## Figures and Tables

**Table 1:** Effects of SI on lipid metabolism and dyslipidemia.

SI Preparation	Dose	Experimental Model	Outcomes	Reference
SI oil	5 or 10 mL for 16 weeks	Healthy adults	↓ TC and LDLc ↑ HDLc	[48]
SI oil	15 mL	Metabolically healthy and unhealthy men given high-fat meal	↓ postprandial TC and IL-6 in metabolically healthy men ↓ postprandial IL-6 in metabolically unhealthy men	[49]
SI oil	5 or 10 mL (contains 2 g or 4 g ω-3/day) for 16 weeks	Hypercholesterolemic patients	↓ TC and LDLc ↑ HDLc	[47]
SI emulsion oil	2.5 mL (contains 0.2 g or 0.5 g ω-3/day) for 8 weeks	Obese rats	↓ TC, TG, and LDLc ↑ HDLc	[46]
SI oil	0.5–1.5 mL/kg for 8 weeks	Obese rats	↓ TC, TG, and LDLc Reverse gut microbiota dysbiosis and metabolome Improve bile acid compositions	[59]
SI nutshell, baby nut and leaf hot water extracts SI nut oil	125 µg/mL	In vitro enzyme inhibitory assays	Only SI nutshell hot water extract ↓ HMG-CoA reductase and cholesterol esterase activities	[53]

Abbreviations: ↑, increase; ↓, decrease; HDLc, high-density lipoprotein cholesterol; HMG-CoA, 3-hydroxy-3-methylglutaryl-coenzyme; IL-6, interleukin-6; LDLc, low-density lipoprotein cholesterol; TC, total cholesterol.

**Table 2:** Effects of SI on obesity.

SI Preparation	Dose	Experimental Model	Outcomes	Reference
SI oil emulsion	2.5 mL (contains 0.25 g and 0.5 g ω-3/day) for 8 weeks	Obese rats	↓ MDA and AOPP ↑ catalase activity ↓ IL-6 and TNF-α ↓ leptin ↑ adiponectin ↑ PPAR-α	[46]
SI oil	0.5–1.5 mL/kg for 8 weeks	Obese rats	↓ mean adipocyte size ↓ hepatic steatosis, hepatic lipase activity and inflammation ↓ PGS1 expression ↑ lipolysis	[59]
SI meal-derived peptides	0.1–0.5 mM	In vitro enzyme inhibitory assay Oleic acid-induced HepG2 cells	↓ pancreatic lipase activity ↓ intracellular fat accumulation and ROS levels in HepG2 cells	[68]
SI husk aqueous ethanol extract	0.4 mg/mL	In vitro enzyme inhibitory assay	↓ lipase activity	[70]

Abbreviations: ↑, increase; ↓, decrease; AOPP, advanced protein oxidation products; IL-6, interleukin-6; MDA, malondialdehyde; PPAR-α, peroxisome proliferator-activated receptor alpha; PGS1, phosphatidylglycerol phosphate synthase 1; TNF-α, tumor necrosis factor alpha.

**Table 3:** Effects of SI on blood glucose and diabetes.

SI Preparation	Dose	Experimental Model	Outcomes	Reference
SI oil	15 mL	Healthy adults given high-fat breakfast	↓ postprandial hyperglycemia ↑ insulin sensitivity ↑ SIRT-1 expression in healthy adults with higher baseline triglycerides and glycemic response	[108]
SI leaves water extract	400 mg/kg for 6 weeks	Type 1 diabetic rats	↓ FBS ↑ insulin sensitivity and glucose tolerance ↓ gut microbiota dysbiosis	[107]
SI oil	0.5–2 mL/kg for 5 weeks	Type 2 diabetic rats	↓ FBS ↑ insulin sensitivity indices and glucose tolerance ↑ IRS-1 and Akt ↓ IR-β ↓ G-6-Pase and PCK-1 activities ↑ hepatic glycogen content ↓ AST and ALT ↓ MDA ↑ SOD, CAT and GPX activities ↓TNF-α and IL-6	[98]
SI husk and shell aqueous ethanol extract	0.025 mg/mL	In vitro enzyme inhibitory assays	↓ α-glucosidase and α-amylase activities	[70]
SI essential oil	25 µg/mL	In vitro enzyme inhibitory assays	↓ α-amylase activity	[86]
SI meal-derived peptides	0.25–0.5 mM	In vitro enzyme inhibitory assay Palmitic acid-induced insulin resistant HepG2 cells	↓ DPP-IV activity ↑ glucose consumption by HepG2 cells	[91]

Abbreviations: ↑, increase; ↓, decrease; Akt, protein kinase B; ALT, alanine transaminase; AST, aspartate transaminase; CAT, catalase; DPP-IV, dipeptidyl peptidase IV; FBS, fasting blood sugar; G-6-Pase, glucose-6-phosphatase; GPX, glutathione peroxidase; IR-β, insulin receptor-β; IRS-1; insulin receptor substrate-1; IL-6, interleukin-6; MDA, malondialdehyde; PCK-1, phosphoenolpyruvate carboxykinase-1; SIRT-1, sirtuin-1; SOD, superoxide dismutase; TNF-α, tumor necrosis factor alpha.

**Table 4:** Effects of SI on blood pressure and hypertension.

SI Preparation	Dose	Experimental Model	Outcomes	Reference
SI oil	10 or 15 mL for 4 months	Healthy adults	↓ SBP and DBP ↓ LDLc	[48]
SI shell extract	400 mg/kg for 51 days	SHR and WKY rats on high-salt diet	↓ SBP ↓ LTCC expression ↑ Na+/K+-ATPase expression Restored calcium and sodium homeostasis ↓ MDA ↑ SOD and GSH ↑ eNOS expression ↑ NO ↑ 5-MTHF Reshaped gut microbiota and metabolome, ↑ prevalence of Roseburia and dihydrofolic acid Normalized F/B ratio	[122]
SI protein hydrolysates	98 µg/mL	In vitro enzyme inhibitory assay	↓ ACE activity	[118]
SI husk and shell aqueous ethanol extract	0.013 mg/mL	In vitro enzyme inhibitory assay	↓ ACE activity	[70]

Abbreviations: ↑, increase; ↓, decrease; 5-MTHF, 5-methyltetrahydrofolate; ACE, angiotensin-converting enzyme; DBP, diastolic blood pressure; eNOS, endothelial nitric oxide synthase; F/B, Firmicutes/Bacteroidetes; GSH, glutathione; LDLc, low-density lipoprotein cholesterol; LTCC, L-type calcium channels; MDA, malondialdehyde; NO, nitric oxide; SBP, systolic blood pressure; SOD, superoxide dismutase.

## Data Availability

Data sharing is not applicable.
